# High- vs. Low-Intensity Statin Therapy and Changes in Coronary Artery Calcification Density after One Year

**DOI:** 10.3390/jcm12020476

**Published:** 2023-01-06

**Authors:** Lukas Hermann Vogel, Iryna Dykun, Paolo Raggi, Axel Schmermund, Tienush Rassaf, Amir Abbas Mahabadi

**Affiliations:** 1 West German Heart and Vascular Center, Department of Cardiology and Vascular Medicine, University Hospital Essen, 45147 Essen, Germany; 2 Department of Medicine Division of Cardiology, Alberta Heart Institute, University of Alberta, Edmonton, AB T6G 2R3, Canada; 3 Cardioangiological Center Bethanien, CCB, 60431 Frankfurt am Main, Germany

**Keywords:** statin therapy, Agatston score, coronary artery calcification, CAC density

## Abstract

Background: Statin therapy promotes the progression of coronary artery calcification (CAC). Comparing patients on high (HIST) vs. low-to-intermediate intensity statin therapy (LIST), randomized controlled trials with a one-year follow-up failed to document a relevant difference in the Agatston score and CAC volume. We evaluated whether statin intensity modifies CAC density at one year. Methods: We performed a pooled analysis of two randomized-controlled trials (BELLES, EBEAT), comparing the effects of HIST (Atorvastatin 80 mg) vs. LIST (Pravastatin 40 mg, Atorvastatin 10 mg) on CAC measures after one year. The differences in CAC density and its change were compared using the two-sided *t*-test. Results: Data from 852 patients (66.7% female) with available baseline and follow-up CT were evaluated from both trials. HIST vs. LIST more effectively reduced LDL-cholesterol (annualized change: −45.8 ± 38.5 vs. −72.9 ± 46.0 mg/dL, *p* < 0.001). Mean CAC density increased from 228.8 ± 35.4 HU to 232.6 ± 37.0 HU (*p* < 0.0001) at one-year follow-up. Comparing patients on HIST vs. LIST, CAC density at follow-up (HIST: 231.9 ± 36.1 HU vs. LIST: 233.3 ± 37.7 HU, *p* = 0.59) and its change from baseline (HIST: 4.0 ± 19.1 HU vs. LIST: 3.6 ± 19.6 HU, *p* = 0.73) did not differ. Subgroup analyses, stratifying by LDL reduction (<median: 2.0 ± 24.3 HU, ≥median: 3.6 ± 21.9 HU, *p* = 0.34), Agatston score at baseline (<100: 2.6 ± 22.5 HU vs. 3.2 ± 25.6 HU, *p* = 0.82; ≥100: 4.8 ± 17.0 HU vs. 3.8 ± 16.6 HU, *p* = 0.44, for HIST vs. LIST; respectively), and equal number of lesions in both CT scans (3.7 ± 20.3 HU vs. 7.0 ± 22.2 HU, *p* = 0.24) showed similar results. Conclusion: HIST vs. LIST leads to a higher reduction in cholesterol levels, which does not translate into relevant differences in the change of CAC density at one-year follow-up.

## 1. Introduction

There is a well-established relationship between low-density lipoprotein cholesterol (LDL-C) levels and risk of cardiovascular events [1]. High-dose statin therapy (HIST) halts coronary plaque progression [2]. In addition, when achieving LDL levels of <70 mg/dL, statin therapy has the ability to reduce plaque burden, as documented in randomized controlled trials using intravascular ultrasound (IVUS) of the coronary arteries [2,3]. Independently of their plaque-regressive effects, IVUS data found statin therapy to induce coronary atheroma calcification [4].

Computed tomography (CT)-derived coronary artery calcification (CAC), as quantified by the Agatston score, is widely used as a surrogate marker of atherosclerotic burden in the coronary arteries and associates with cardiovascular events [5,6,7]. In mid- to long-term follow-up, statin therapy increases the CAC score as documented by serial non-contrast cardiac CT, suggesting that the plaque-stabilizing effect of statins may be reflected in a higher CAC score [8,9]. However, with a one-year follow-up, randomized controlled trials failed to detect an influence of HIST vs. low-to-intermediate-intensity statin therapy (LIST) on the progression of the Agatston and CAC volume score [10,11,12,13].

Coronary plaques with lower density including spotty calcifications may represent dynamic and early stages of atherosclerosis [14]. Likewise, investigators from the Multi-Ethnic Study of Atherosclerosis (MESA) described that CAC density was inversely related with cardiovascular disease risk [15,16]. These findings call for additional data, evaluating the effect of statin therapy on CAC density. Therefore, the rationale for this pooled analysis of individual patient data from two randomized controlled trials was to assess whether HIST, as compared LIST, would alter CAC density after one year.

## 2. Materials and Methods

### 2.1. Study Population

This meta-analysis includes data from two prospective, randomized, double-blind studies, the BELLES [11] and the EBEAT [10] trial (Supplementary Figure S1). Both studies were designed to examine potential effects of a one-year statin therapy on CAC changes. The BELLES trial included hypercholesterolemic postmenopausal mostly white women recruited in 96 US sites, treated with either Pravastatin 40 mg or Atorvastatin 80 mg [11]. The EBEAT trial included mostly men (75%), treated with either Atorvastatin 10 mg or 80 mg [10].

Both studies were designed to evaluate the influence of statin intensity on the changes of the CAC volume score. Patient-level data from both trials were stratified by intensity of statin therapy, as classified by AHA/ACC recommendations [17]. This resulted in one group of high intensity statin therapy (HIST, Atorvastatin 80 mg) and a second group of low-to-intermediate intensity statin therapy (LIST, Pravastatin 40 mg or Atorvastatin 10 mg). All patients with available baseline and follow-up CT scans and information on CAC density, Agatston score, lesion volume score, and the number of lesions were included. Patients with zero lesions at baseline (n = 4) or a mean lesion density below 130 HU (baseline CT scan: n = 12, follow-up CT scan: n = 5) were excluded. Patients with missing information regarding cholesterol or triglyceride levels at baseline or follow-up were excluded for the performed subgroup analysis. We calculated that a sample size of 722 patients would be sufficient to detect a difference in the change of CAC density between both groups of ≥2.5 HU (standard deviation of 12 HU) at 80% power (5% type 1 error rate) with 1:1 sampling ratio.

### 2.2. CAC Quantification

Details for the assessment of serial CAC measurements for each study have been described previously [10,11]. Briefly the BELLES trial pooled electron-beam computed tomography (EBCT) data of 35 sites all using C-150 Imatron scanners (GE/Imatron, California USA) with a standardized protocol. During a single breath 36–40 slices of 3 mm were obtained in a 100 ms scanning time and triggered at 60% RR interval. Time between baseline and follow-up EBCT was 52.0 ± 8.2 weeks.

The EBEAT trial used EBCT in nine different centers running a standardized protocol [10]. High-resolution EBCT in single-slice mode with continuous, non-overlapping slices of 3 mm thickness was applied with an acquisition time of 100 ms in a 26 cm^2^ field of view. Scans were triggered at 80% of RR interval and patients were asked to hold their breath. Follow-up EBCT was performed 54.8 ± 7.8 weeks after baseline examination. For the current analysis, we included information on the overall CAC volume, density, and the Agatston score, as well as the number of calcified lesions at baseline and follow-up.

### 2.3. Statistical Analysis

Continuous variables are reported as mean ± standard deviation (SD) when normally distributed and median (interquartile range: IQR) when non-normally distributed. Discrete variables are given in frequency and percentages. Lipid levels and CAC measures as well as their absolute change were compared in patients with HIST vs. LIST using a 2-sided *t*-test or a Mann–Whitney U test (for non-normal distributed variables). The correlation between baseline and follow-up CAC density was evaluated using the Pearson correlation coefficient. A subgroup analysis was performed in patients with baseline Agatston score of < vs. ≥100. As the primary endpoint of the present meta-analysis is based on an intention to treat analysis of the two underlying trials, we performed another subgroup analysis, stratifying patients into LDL reduction of ≥median and <median to evaluate whether potential discontinuation of the therapy or changes in lipid lowering strategies may have biased our results. To rule out that new calcified lesions may have influenced the results, we performed a sensitivity analysis, only including cases with identical numbers of lesions at baseline and follow-up.

All data analyses were performed using the SPSS (IBM, version 27) as well as R Studio (version 1.4.1103). Data from both studies were pooled using the Revman 5.3 software (The Cochrane Collaboration). A *p*-value of < 0.05 indicated statistical significance.

## 3. Results

Table 1 describes baseline characteristics for both studies included in this analysis (n = 852). The BELLES cohort (n = 476) included only female subjects with a mean age of 65.1 ± 6.2 years. The EBEAT trial included 376 patients with a mean age of 61.5 ± 8 years, of which 25% were women. Table 2 describes baseline and follow-up lipid levels at follow-up, where total cholesterol and LDL-cholesterol levels were lower in the HIST group.

**Figure 1:**
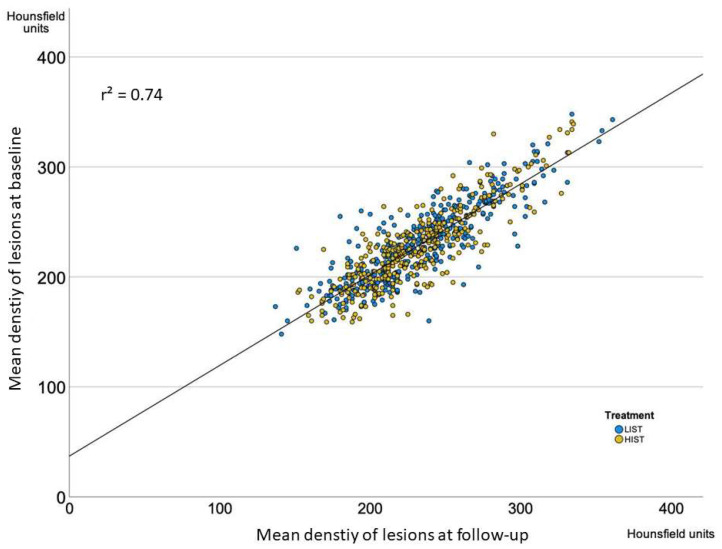
Correlation of the absolute lesion density at baseline and follow-up CT-scan, stratified by treatment group (LIST (blue) vs. HIST (yellow)) of the cohort of the BELLES and the EBEAT trials.

In the overall cohort, all measured CAC parameters were higher after one year (CAC density: 228.8 ± 35.4 vs. 232.6 ± 37.0; *p* < 0.0001; Agatston score: 170.9 (79.5, 387.1) vs. 214.8 (94.6, 465.9); *p* < 0.0001 ^NP^; Volume score: 135.6 (63.6, 313.4) vs. 168.4 (78.5, 370.1) *p* < 0.0001 ^NP^; number of lesions: 6 (3,10) vs. 7 (4,12); *p* < 0.0001 ^NP^, at baseline and follow-up, respectively). Table 3 depicts mean calcified density, the Agatston score, the CAC volume score, and the number of calcified lesions at baseline and follow-up, as well as their absolute changes in both treatment groups. There were no major differences regarding the change in any EBCT-derived CAC measurements at baseline and one-year follow-up in the HIST vs. LIST group. Figure 1 depicts the correlation between the calcification density at baseline and follow-up in both treatment groups, confirming that the overall high correlation between the density in serial measures was not different in the HIST vs. LIST group (r² = 0.74).

Table 4 shows the EBCT-derived lesion characteristics stratified according to the relative LDL reduction (< vs. ≥median (−62 mg/dL)), where again no significant difference in the change from baseline was noted. To gain further insights we executed different subgroup analyses. For comparison of early and later stages of CAC, we divided the cohort by baseline Agatston score < vs. ≥100. Again, stratifying by patients with HIST vs. LIST, we found no relevant difference in CAC density in any group (Supplementary Table S1). The subgroup analysis including only patients with identical numbers of lesions at baseline and follow-up, further confirmed no difference in CAC density and other CAC measures in the HIST vs. LIST group (Supplementary Table S2) at one year.

## 4. Discussion

In clinical practice, CAC scoring is used to identify patients at increased risk for future cardiovascular events [5,18]. In addition to traditional cardiovascular risk factors, CAC scoring enables identification of patients with the need for intensified risk factor modification including statin therapy [7,19,20]. Likewise, in primary prevention, only patients with the presence of CAC are likely to benefit from statin initiation [21]. Currently, effectiveness of statin therapy is monitored by serial laboratory assessments, while imaging-derived markers of cardiovascular risk are limited [19]. For CAC, its progression along with the individual’s age and gender specific percentile was found to follow a predictable pattern [22]. Moreover, rapid progression above the expected range of CAC progression is associated with increased event rates [23]. With 5 years of follow-up, the population-based Heinz Nixdorf Recall study found that statin therapy is independently associated with rapid CAC progression [8]. Likewise, in a post-hoc analysis of the St. Francis trial with a median follow-up of 4 years, CAC changes were higher in statin treated groups [9]. In contrast, randomized controlled trials with one-year follow-up did not find an effect of intensified lipid lowering therapy on the CAC progression [10,11,13]. Apart from the CAC volume score and the Agatston score, CAC density as an additional measure of coronary artery calcification has gained interest due to its inverse correlation with cardiovascular events [15], reflecting early and potentially vulnerable lesions [14]. As data on serial coronary IVUS imaging demonstrated statin therapy to stabilize these spotty lesions by increasing its calcification at 18 to 24 months follow-up [4], we evaluated whether CAC density may be an early non-invasive imaging marker of plaque stabilization following HIST. In this pooled analysis of two prospective randomized trials, we observed an expected overall progression of CAC but did not demonstrate differences in the effect of HIST vs. LIST on measured CAC density, number of lesions, or the Agatston or volume scoring after a one-year period. To account for (a) different effects of applied lipid-lowering therapy, (b) early or later stages of subclinical coronary artery disease, and (c) new onset of calcified lesions, we performed several subgroup and sensitivity analyses. However, we did not find any difference in all investigated CAC measures in any of these subgroups. Further research on other non-invasive imaging techniques, enabling quantification and characterization of the overall coronary plaque burden such as contrast-enhanced CT coronary angiography are needed to further evaluate the influence of intensified lipid lowering therapy on coronary atherosclerosis.

The present analysis is based on individualized patient data from two randomized controlled, double-blind clinical trials with inclusion of over 850 patients. The high standardization of both trials as well as identical measures for quantification of CAC are a strength of the present work. However, this analysis has several limitations. Most importantly, the duration of follow-up of one year is relatively short. As studies on longer follow-ups demonstrated that statin therapy led to higher CAC progression, further research is warranted, evaluating the long-term effects of lipid lowering therapy on CAC density. Moreover, both studies compared HIST vs. LIST. Therefore, the effect of statin therapy as compared to placebo on CAC density cannot be evaluated by our results. In addition, we cannot disregard unmeasured cofounding that may have biased the results, potentially caused by differences in inclusion/exclusion criteria in each trial. As such, the BELLES trial only included female patients. In contrast, the EBEAT trial included predominantly male subjects. However, we found stable effects that were confirmed in all investigated sensitivity and subgroup analyses.

## 5. Conclusions

After one year of treatment, HIST as compared to LIST leads to a higher reduction in cholesterol levels, which does not translate into a relevant difference in the change of lesion’s density when using serial non-contrast enhanced CT scans of the heart.

## Figures and Tables

**Table 1:** Baseline characteristics for each trial.

	Raggi et. al. BELLES [11] (n = 476)	Schmermund et. al. EBEAT [10] (n = 376)
	Demographics	
Age, years	65.1 ± 6.2	61.5 ± 8 *
Sex (female), n (%)	476 (100)	92 (24.5)
BMI, m/kg^2^	28.9 ± 5.6	27.6 ± 3.7
SBP > 130 mmHg, n (%)	209 (43.9)	237 (63.0)
Smoking status, n (%)		
none	190 (39.9)	88 (23.4)
current	191 (40.1)	100 (26.6)
former	95 (20.0)	188 (50.0)
	Medication and CT-data	
Time between CT-scans, weeks	52.0 ± 8.2	54.8 ± 7.8
HIST, n (%)	218 (45.8)	176 (46.8)

* Data derived from original publication HIST: high intensity statin therapy; BMI: body mass index; SBP: systolic blood pressure; CT: computer tomography.

**Table 2:** Baseline, follow-up, and change in lipid values in low-to-intermediate vs. high intensity statin therapy groups.

	LIST	HIST	*p*-Value
	Total cholesterol		
Baseline, mg/dL	246.2 ± 46.6	245.8 ± 46.8	0.9
Follow-up, mg/dL	200.5 ± 36.9	168.8 ± 40.2	<0.001
Change from baseline, mg/dL	−45.7 ± 41.9	−77.0 ± 51.8	<0.001
	LDL-cholesterol		
Baseline, mg/dL	164.0 ± 40.4	163.9 ±39.8	0.96
Follow-up, mg/dL	118.3 ± 30.8	91.0 ± 35.3	<0.001
Change from baseline, mg/dL	−45.8 ± 38.5	−72.9 ± 46.0	<0.001
	HDL-cholesterol		
Baseline, mg/dL	54.1 ± 13.9	52.9 ± 13.7	0.25
Follow-up, mg/dL	56.5 ± 14.2	54.7 ± 13.5	0.08
Change from baseline, mg/dL	2.4 ± 7.9	1.8 ± 8.3	0.29
	Triglycerides		
Baseline, mg/dL	156 (117, 214)	179 (118, 227)	0.17
Follow-up, mg/dL	137.5 (100, 188.3)	118 (87, 169)	<0.001
Change from baseline, mg/dL	−22.3 ± 74.6	−48.4 ± 94.0	<0.001

LIST: low-to-intermediate-intensity statin therapy; HIST: high-intensity statin therapy; HDL: High-density lipoprotein; LDL: Low-density lipoprotein.

**Table 3:** Baseline, follow-up, and change in EBCT-derived lesion characteristics in patients with high intensity (HIST) and low-to-intermediate intensity statin therapy (LIST).

	LIST (n = 458)	HIST (n = 394)	*p*-Value
	Densitiy of lesions		
Baseline, HU	229.7 ± 35.3	227.8 ± 35.5	0.45
Follow-up, HU	233.3 ± 37.7	231.9 ± 36.1	0.59
Change from baseline, HU	3.6 ± 19.6	4.0 ± 19.1	0.73
	Agatston score		
Baseline	187.7 (82.4, 414,6)	154.5 (75.6, 327.0)	0.16 ^NP^
Follow-up	238.2 (105.8, 482.3)	184.8 (89.3, 416.6)	0.10 ^NP^
Change from baseline	53.4 ± 163.8	58.2 ± 180.2	0.68
	CAC volume score		
Baseline, mm³	146.4 (66.8, 350.7)	184.2 (81.8, 426.0)	0.13 ^NP^
Follow-up, mm³	180.9 (80.5, 404.2)	229.5 (103.8, 516.2)	0.88 ^NP^
Change from baseline, mm³	41.4 ± 130.0	43.9 ± 111.2	0.78
	Number of lesions		
Baseline, n	6 (4, 11)	6 (3, 10)	0.27 ^NP^
Follow-up, n	7 (4, 12)	7 (4, 11)	0.29 ^NP^
Change from baseline, n	0.9 ± 3.6	0.8 ± 3.2	0.75

LIST: low-to-intermediate-intensity statin therapy; HIST: high-intensity statin therapy; HU: Hounsfield units; CAC coronary artery calcification. ^NP^ non-parametric.

**Table 4:** Baseline, follow-up, and change in EBCT-derived lesion characteristics depending on LDL reduction of < vs. ≥median (−62 mg/dL).

	LDL-Reduction ≥ Median (n = 357)	LDL-Reduction < Median (n = 360)	*p*-Value
HIST, n (%)	218 (61.1)	111 (30.1)	-
	Densitiy of lesions		
Baseline, HU	225.9 ± 33.6	230.8 ± 38.4	0.38
Follow-up, HU	229.5 ± 37.4	232.8 ± 44.7	0.29
Change from baseline, HU	3.6 ± 21.9	2.0 ± 24.3	0.35
	Agatston score		
Baseline	149.2 (69.9, 348.1)	184.2 (85.2, 451.7)	0.036 ^NP^
Follow-up	180.8 (83.5, 420.5)	238.6 (103.8, 530.0)	0.032 ^NP^
Change from baseline	47.0 ± 120.0	65.6 ± 146.7	0.063
	CAC volume score		
Baseline, mm^3^	117.8 (58.6, 275.7)	154.1 (69.4, 357.8)	0.014 ^NP^
Follow-up, mm^3^	142.6 (68.4, 338.6)	184.9 (83.3, 415.8)	0.020 ^NP^
Change from baseline, mm^3^	36.4 ± 92.8	51.0 ± 115.3	0.062
	Number of lesions		
Baseline, n	6 (3, 10)	7 (4, 12)	0.017 ^NP^
Follow-up, n	7 (3, 11)	8 (4, 13)	0.005 ^NP^
Change from baseline, n	0.5 ± 3	1.0 ± 3.6	0.071

HIST: high-intensity statin therapy; HU: Hounsfield units; CAC: coronary artery calcification. ^NP^ non-parametric.

## Data Availability

Not applicable.

## Supplementary Materials

The following supporting information can be downloaded at: https://www.mdpi.com/article/10.3390/jcm12020476/s1.
