# Supplementary material for: High- vs. Low-Intensity Statin Therapy and Changes in Coronary Artery Calcification Density after One Year
Source: J Clin Med. 2023 Jan 6;12(2):476. doi: 10.3390/jcm12020476 (PMC9867203; doi:10.3390/jcm12020476)
Supplement: Supplementary file 1 [file jcm-12-00476-s001.zip › jcm-2105893-supplementary.pdf]

Supplementary Table S1. Baseline, follow-up, and change in EBCT-derived lesions characteristics in subgroup analyses, depending on initial Agatston score (<vs. ≥100)

	Agatston Score at baseline <100			Agatston Score at baseline ≥100		
	**LIST (n=133)**	**HIST (n=136)**	**p-Value**	**LIST (n=325)**	**HIST (n=258)**	**p-Value**
	Densitiy of lesions					
Baseline, HU	211.7 ± 35.3	215.7 ± 37.2	0.37	237.0 ± 32.6	234.2 ± 32.8	0.31
Follow-up, HU	214.9 ± 40.4	218.2 ± 34.1	0.46	240.8 ± 33.8	239.1 ± 35.1	0.55
Change from baseline, HU	3.2 ± 25.6	2.6 ± 22.5	0.82	3.8 ± 16.6	4.8 ± 17.0	0.44
	Agatston score					
Baseline	58 (42.5, 73.5)	58.2 (42.5, 79.0)	0.53^{NP}	273.5 (171, 604)	269.9 (158, 554)	0.83^{NP}
Follow-up	64.9 (48.0, 98.3)	74.5 (55.5, 93.6)	0.21^{NP}	347.3 (214, 654)	335.9 (184, 636)	0.82^{NP}
Change from baseline	20.1 ± 51.0	19.1 ± 31.0	0.85	67.1 ± 190.1	78.8 ± 218.9	0.49
	CAC volume score					
Baseline, mm^3	46.4 (36.0, 60.5)	47.7 (33.7,63.5)	0.06^{NP}	226.8 (135, 483)	208.0 (119, 430)	0.59^{NP}
Follow-up, mm^3	53.1 (40.3, 79.3)	62.8 (44.4, 79.0)	0.99^{NP}	270.0 (166, 543)	280.3 (146, 538)	0.77^{NP}
Change from baseline, mm^3	15.3 ± 42.7	15.9 ± 23.6	0.89	51.2 ± 144.8	62.7 ± 169.2	0.38
	Number of lesions					
Baseline, n	3 (2.5)	3 (2.6)	0.39 ^{NP}	8 (5.1)	8.5 (5.1)	0.49^{NP}
Follow-up, n	3 (2.5)	4 (2.5)	0.99 ^{NP}	9 (6.1)	9 (6.2)	0.81^{NP}
Change from baseline, n	1 ± 3.8	0.6 ± 2.0	0.29	3.8 ± 16.6	4.8 ± 17.0	0.44

LIST: low-to-intermediate-intensity statin therapy; HIST: high-intensity statin therapy; HU: Hounsfield units; CAC: Coronary artery calcification.
^{NP} non-parametric.

Supplementary Table S2. Baseline, follow-up, and change in EBCT-derived lesions characteristics in subgroup analysis, only including patients with identical number of lesions per vessel at baseline and follow-up.

	**LIST (n=117)**	**HIST (n=108)**	**p-Value**
	Densitiy of lesions		
Baseline, HU	229.6 ± 34,7	228.6 ± 35.2	0.83
Follow-up, HU	236.6 ± 37.1	232.3 ± 35.5	0.37
Change from baseline, HU	7.0 ± 22.2	3.7 ± 20.3	0.24
	Agatston score		
Baseline	119.5 (58.9, 206.2)	108.1 (63.2, 202.7)	0.74^{NP}
Follow-up	138.8 (63.5, 294.7)	125.1 (71.4, 252.1)	0.55^{NP}
Change from baseline	214.5 ± 275.4	201.9 ± 268.9	0.60
	CAC volume score		
Baseline, mm^3	102 (48.8, 170.2)	88.8 (46.9, 171.5)	0.74^{NP}
Follow-up, mm^3	109.0 (49,6, 228.5)	98.3 (56.5, 211.4)	0.95^{NP}
Change from baseline, mm^3	28.1 ± 68.5	22.8 ± 55.0	0.65
	Number of lesions		
Baseline, n	5.3 ± 4.7	4.9 ± 4.2	0.98^{NP}
Follow-up, n	5.3 ± 4.7	4.9 ± 4.2	0.98^{NP}
Change from baseline, n	0	0	---

LIST: low-to-intermediate-intensity statin therapy; HIST: high-intensity statin therapy; HU: Hounsfield units; CAC coronary artery calcification.
^{NP} non-parametric.
